# *ALDH1A1* Genetic Variations May Modulate Risk of Parkinson’s Disease in Han Chinese Population

**DOI:** 10.3389/fnins.2021.620929

**Published:** 2021-03-19

**Authors:** Hui-Hui Fan, Qing Guo, Jing Zheng, Yi-Zhi Lian, Shi-Shi Huang, Yue Sun, Ming Zou, Jian-Hong Zhu, Xiong Zhang

**Affiliations:** ^1^Department of Preventive Medicine, Wenzhou Medical University, Wenzhou, China; ^2^Department of Geriatrics and Neurology, The Second Affiliated Hospital and Yuying Children’s Hospital, Wenzhou Medical University, Wenzhou, China

**Keywords:** *ALDH1A1*, Parkinson’s disease, single nucleotide polymorphism, rs7043217, genetic association

## Abstract

**Background:** Studies in animal models have suggested that aldehyde dehydrogenase 1 (encoded by *ALDH1A1*) protects against Parkinson’s disease (PD) by reducing toxic metabolites of dopamine. Herein we aimed to investigate whether *ALDH1A1* was genetically associated with PD susceptibility in humans.

**Methods:** A Han Chinese population of 1,039 subjects was recruited to analyze six tag-single nucleotide polymorphisms (SNPs), followed by haplotype analyses and variants interaction analyses. Real-time PCR was used to analyze mRNA levels of *ALDH1A1* in peripheral blood of 42 subjects.

**Results:** The tag-SNP rs7043217 of *ALDH1A1* was significantly associated with PD susceptibility with the T serving as a risk allele (genotype frequency, *P* = 0.030; allele frequency, *P* = 0.013, OR = 1.258, 95% CI = 1.050–1.508). Multiple haplotypes were linked to abnormalities of PD risk, topped by a 4-SNP GGTA module in the order of rs4646547, rs1888202, rs7043217, and rs647880 (*P* = 9.610 × 10^–8^, OR = 6.420, 95% CI = 2.944–13.998). Interaction analyses showed that a simultaneous presence of the CC genotype of rs7043217 and the TT genotype of *ALDH2* variant rs4767944 conferred an elevated protection against PD (*P* = 4.68 × 10^–4^, OR = 0.378, 95% CI = 0.219–0.652). The mRNA expression of *ALDH1A1* showed a trend of reduction (*P* = 0.084) in PD patients compared to the controls.

**Conclusion:** Our results provide novel genetic insights into the role of ALDH1 in PD pathogenesis.

## Introduction

Parkinson’s disease (PD) is mainly of sporadic origin and the second most common neurodegenerative disorder characterized by the progressive loss of dopaminergic neurons in the substantia nigra pars compacta (SNpc). Besides familial PD cases which are caused by monogenic mutations such as in *SNCA* and *LRRK2*, the etiology of sporadic PD remains largely unknown but is considered to be influenced by both genetic factors and environmental exposure (Kalia and Lang, 2015).

Aldehyde dehydrogenase (ALDH) is a family of enzymes that catalyze the oxidation of aldehydes to their corresponding carboxylic acids. In dopaminergic neurons, ALDH converts the dopamine metabolite 3,4-dihydroxyphenylacetaldehyde (DOPAL) into 3,4-dihydroxyphenylacetic acid (DOPAC), which is further metabolized to form homovanillic acid (Grunblatt and Riederer, 2016; Masato et al., 2019). DOPAL is neurotoxic by triggering α-synuclein aggregates, thus provoking the loss of dopaminergic neurons (Marchitti et al., 2007; Burke et al., 2008). It has been shown that benomyl, a pesticide, inhibits ALDH activity in rat primary cells of SNpc, leading to increased DOPAL production and cytotoxicity (Fitzmaurice et al., 2013).

ALDH has a number of isoforms. Amidst, ALDH1 is cytosolic and highly expressed in dopaminergic neurons. ALDH2 is mitochondrion-located and moderately expressed in these neurons (Cai et al., 2014). Genetic studies of PD patients indicate that a clade of *ALDH2* haplotypes by pesticide exposure exacerbates predisposition to this disease (Fitzmaurice et al., 2014), and the rs4767944 variant of *ALDH2* is associated with PD risk in Han Chinese (Zhang et al., 2015). On the other hand, ALDH1-positive dopaminergic neurons are reported to be more resistant than the negative ones to α-synuclein-mediated cytotoxicity in mouse SNpc (Liu et al., 2014). Moreover, ALDH1 mRNA and protein are found to be down-regulated in the substantia nigra of post-mortem PD patients (Galter et al., 2003; Grunblatt et al., 2004; Werner et al., 2008). ALDH1 is highly expressed in dopaminergic neurons (Cai et al., 2014) and appears to be the pivotal isoform of ALDH in protection against PD. Nonetheless, an important question to be answered is whether *ALDH1A1* is genetically associated with PD in humans. A Han Chinese cohort was thus recruited to address this hypothesis by analyzing six tag-single nucleotide polymorphisms (SNPs) of *ALDH1A1*.

## Materials and Methods

### Subjects

A total of 1,039 Han Chinese were included for tag-SNP analysis from eastern China, including 506 sporadic PD patients (259 males and 247 females) and 533 controls (282 males and 251 females). The median age of the patients and controls was 67 (interquartile range, 60–74) and 58 (interquartile range, 50–68), respectively. For quantitation of *ALDH1A1* mRNA levels, another 19 sporadic PD patients (10 males and nine females; median age, 64, interquartile range, 58–68) and 23 control subjects (11 males and 12 females; median age, 59, interquartile range, 54–70) were recruited. PD patients were diagnosed by two neurologists according to the United Kingdom Parkinson’s Disease Society Brain Bank Criteria (Hughes et al., 1992). Patients with a family history of PD, or with secondary and atypical Parkinsonism were excluded. Control subjects were free of neurological disorders determined by medical history, physical and auxiliary laboratory examinations of blood routine, blood lipid, blood sugar, liver, and kidney functions. Informed written consents were obtained from all participants. The study was approved by the Ethics Committee of The Second Affiliated Hospital and Yuying Children’s hospital, Wenzhou Medical University (2017-31).

### Tag-SNPs and Genotyping

Six variants of *ALDH1A1* tag-SNP, including rs4646547 (T > G), rs1888202 (G > C), rs348471 (C > T), rs7043217 (C > T), rs647880 (A > G), and rs8187876 (G > A), were selected using Haploview v.4.2 as described previously (Zhang et al., 2015). The construction criteria were minor allele frequency (MAF) ≥ 0.1 and *r*^2^ > 0.8 (Supplementary Figure 1).

Genomic DNA was extracted from peripheral blood samples using TIANamp Genomic DNA Kit (Tiangen, Beijing, China) according to the manufacturer’s instruction. Five of the tag-SNPs (rs4646547, rs348471, rs7043217, rs647880, and rs8187876) were analyzed by the polymerase chain reaction-restriction fragment length polymorphism (PCR-RFLP). The rs1888202 variant was analyzed by Sanger sequencing due to a lack of restriction enzyme recognition site. Primers and restriction enzymes (New England Biolabs, Ipswich, MA, United States) were detailed in Supplementary Table 1.

### RNA Extraction and Real-Time PCR

Total RNA was extracted using RNAiso plus (Takara, Shiga, Japan). An amount of 400 ng RNA was used for synthesis of the first-strand cDNA by PrimeScript RT reagent Kit (Takara, Shiga, Japan). PCR amplification was performed using ChamQ Universal SYBR qPCR Master Mix (Vazyme, Nanjing, China) with the following conditions: 95°C for 10 min, 40 cycles of 95°C for 10 s, 60°C for 15 s, and 72°C for 20 s, and a final extension of 72°C for 10 min. Primers were detailed in Supplementary Table 1. The mRNA levels were expressed as 2^–Δ*CT*^ following normalization to their respective *ACTB*.

### Statistical Analysis

Statistical analyses were performed using the Statistical Package for Social Science Program (SPSS for Windows, version 23.0). Hardy–Weinberg equilibrium in genotype distribution was assessed using χ^2^ test. Following Kolmogorov-Smirnov test for normality, age difference was evaluated by Mann–Whitney *U* test, and levels of gene expression were expressed as mean ± standard deviation and analyzed by student’s *T*-test. The difference in genotype and allele frequencies, and the interaction between genes were analyzed using logistic regression model with gender and age as covariates. The χ^2^ test was used to assess differences in gender, and haplotype frequencies between the PD cases and controls. The haplotype analysis was not adjusted for gender and age, because individual haplotypes are unable to be deduced from subjects with two or more heterozygous SNP sites, and thus individual haplotype assignment would induce additional assumptions that are less reliable (Zhang et al., 2004; Woo et al., 2005).

The haplotype construction and association analysis were performed using SHEsis Online Version^1^ (Shi and He, 2005). A backward elimination method was used to identify the highest-risk haplotype for PD (Francis et al., 2007). In brief, all the six tag-SNPs were used to produce the first haplotype model. One SNP was then removed each time to create 5-SNP models. Of these, the one with the lowest associated *P* value was deemed the best model for next round of elimination. As such, the best 4-, 3-, and 2-SNP models were determined. A two-tailed *P* value < 0.05 was considered statistically significant.

## Results

### Associations of the Variant rs7043217 With PD Susceptibility

Genotype distributions of the six tag-SNPs were in accordance with Hardy-Weinberg equilibrium (*P* > 0.05). The PD cases and controls were comparable in gender (*P* > 0.05) but different in age (*P* < 0.05). Results showed that genotype and allele frequencies of rs4646547, rs1888202, rs647880, and rs8187876 were comparable (*P* > 0.05) between the PD cases and controls (Table 1). The rs348471 significantly differed (*P* = 0.045) in genotype but not in allele frequency. In contrast, significant differences were found in both genotype (*P* = 0.030) and allele (*P* = 0.013) frequencies of rs7043217 between the cases and controls, with the T allele serving as a risk allele for PD (OR = 1.258, 95% CI = 1.050–1.508). Analyzing of rs7043217 using three genetic models (additive, dominant, and recessive) showed that rs7043217 was significantly associated with PD in the dominant model (*P* = 0.009; Table 2) and additive model (*P* = 0.015; Table 2), further suggesting that the T allele was a risk allele for PD.

**TABLE 1:** Genotype and allele frequencies of the six *ALDH1A1* tag-SNPs in PD patients and controls.

Tag-SNPs	Genotype, *n* (%)			*P*^a^	Allele, *n* (%)		*P*^a^	OR (95% CI)
rs4646547	TT	TG	GG		T	G		
Control	103 (19.3)	276 (51.8)	154 (28.9)		482 (45.2)	584 (54.8)		
PD	82 (16.2)	271 (53.6)	153 (30.2)	0.534	435 (43.0)	577 (57.0)	0.564	1.055 (0.880–1.263)
rs1888202^*b*^	GG	GC	CC		G	C		
Control	168 (31.5)	263 (49.3)	102 (19.1)		599 (56.2)	467 (43.8)		
PD	167 (33.0)	254 (50.2)	85 (16.8)	0.797	588 (58.1)	424 (41.9)	0.590	0.951 (0.794–1.140)
rs348471	CC	CT	TT		C	T		
Control	143 (26.8)	288 (54.0)	102 (19.1)		574 (53.8)	492 (46.2)		
PD	149 (29.4)	237 (46.8)	120 (23.7)	0.045*	535 (52.9)	477 (47.1)	0.802	1.023 (0.855–1.225)
rs7043217	CC	CT	TT		C	T		
Control	199 (37.3)	235 (44.1)	99 (18.6)		633 (59.4)	433 (40.6)		
PD	145 (28.7)	250 (49.4)	111 (21.9)	0.030*	540 (53.4)	472 (46.6)	0.013*	1.258 (1.050–1.508)
rs647880	AA	AG	GG		A	G		
Control	135 (25.3)	261 (49.0)	137 (25.7)		531 (49.8)	535 (50.2)		
PD	132 (26.1)	248 (49.0)	126 (24.9)	0.950	512 (50.6)	500 (49.4)	0.813	0.979 (0.818–1.171)
rs8187876	GG	GA	AA		G	A		
Control	149 (28.0)	269 (50.5)	115 (21.6)		567 (53.2)	499 (46.8)		
PD	126 (24.9)	262 (51.8)	118 (23.3)	0.520	514 (50.8)	498 (49.2)	0.278	1.104 (0.923–1.321)

^*a*^Adjusted with age and sex; ^*b*^rs1888202 genotyped by Sanger sequencing, and the other five SNPs genotyped by polymerase chain reaction-restriction fragment length polymorphism; *P < 0.05. CI, confidence interval; OR, odds ratio; PD, Parkinson’s disease; SNP, single nucleotide polymorphism.

**TABLE 2:** Association between rs7043217 and PD using dominant, recessive and additive models.

Model	Genotype	*P*^a^	OR (95% CI)
Dominant	CC vs. CT + TT	0.009**	1.437 (1.095–1.885)
Recessive	CC + CT vs. TT	0.202	1.229 (0.895–1.686)
Additive	CC vs. CT vs. TT	0.015*	1.246 (1.043–1.488)

^*a*^Adjusted with age and sex; *P < 0.05; **P < 0.01. CI, confidence interval; OR, odds ratio; PD, Parkinson’s disease.

A total of 415 PD patients with subtype records were classified into three subtypes, that is, tremor dominant, postural instability/gait difficulty, and indeterminate. Association analysis of the SNPs with PD subtypes showed that the associations were present in the genotype frequency of rs7043217 between patients of the tremor-dominant subtype and the controls (*P* = 0.007), and in the allele frequency of rs7043217 between patients of the postural instability/gait difficulty subtype and the controls (*P* = 0.032, OR = 1.329, 95% CI, 1.025–1.722; Supplementary Table 2).

### Haplotype Analyses of *ALDH1A1* Tag-SNPs

We analyzed whether *ALDH1A1* haplotypes of the tag-SNPs were associated with the risk of PD. Haplotypes were constructed following the order of rs4646547, rs1888202, rs348471, rs7043217, rs647880, and rs8187876, and those with a frequency < 3.0% in both PD patients and controls were excluded. The remaining 10 haplotypes were listed in Table 3. A significant difference (*P* = 2.880 × 10^–11^) in overall haplotype distribution was observed between the PD patients and controls. Four haplotypes, including GGCTAG, GGCCGA, GCCTGG, and GCCCAG, displayed a statistically significant difference between the cases and controls. In particular, the haplotype GGCTAG rendered an increased risk of PD (*P* = 8.540 × 10^–6^, OR = 5.619, 95% CI = 2.397–13.172), while the GCCCAG carriers might be resistant to PD (*P* = 1.400 × 10^–8^, OR = 0.133, 95% CI = 0.059–0.299).

**TABLE 3:** Haplotype analysis of *ALDH1A1* tag-SNPs in PD patients and controls.

Haplotype^a^	Control, *n* (%)	PD, *n* (%)	*P*	OR (95% CI)
GGTCGA	83.0 (7.8)	65.9 (6.5)	0.214	0.806 (0.574–1.133)
GGCTAG	6.4 (0.6)	33.5 (3.3)	8.540 × 10^–6^***	5.619 (2.397–13.172)
GGCCGA	34.1 (3.2)	52.0 (5.1)	0.031*	1.622 (1.041–2.528)
GCCTAG	244.5 (22.9)	263.0 (26.0)	0.139	1.173 (0.950–1.450)
GCCTGG	37.3 (3.5)	20.4 (2.0)	0.033*	0.555 (0.321–0.962)
GCCCAG	49.5 (4.6)	6.7 (0.7)	1.400 × 10^–8^***	0.133 (0.059–0.299)
TGTTAG	34.4 (3.2)	46.1 (4.6)	0.133	1.413 (0.898–2.224)
TGTCAG	60.5 (5.7)	40.6 (4.0)	0.063	0.679 (0.450–1.023)
TGTCGA	210.5 (19.7)	213.3 (21.1)	0.560	1.069 (0.855–1.336)
TGCCGA	37.9 (3.6)	28.9 (2.9)	0.325	0.781 (0.476–1.280)
Total	1066.0 (100)	1012.0 (100)	2.880 × 10^–11^***	

^*a*^Order of the alleles of the haplotypes: rs4646547, rs1888202, rs348471, rs7043217, rs647880, and rs8187876. Haplotypes with frequency < 3% in both PD patients and controls were excluded from the analysis; *P < 0.05; ***P < 0.001. CI, confidence interval; OR, odds ratio; PD, Parkinson’s disease; SNP, single nucleotide polymorphism.

We further analyzed the highest-risk haplotype of *ALDH1A1* toward PD using backward elimination models. Results showed that the strongest PD-associated haplotype was the GGTA in order of rs4646547, rs1888202, rs7043217, and rs647880 from the best 4-SNP model (*P* = 9.610 × 10^–8^, OR = 6.420, 95% CI = 2.944–13.998; Table 4). The frequencies of the GGTA were 0.7 and 4.4%, respectively, in the controls and PD patients.

**TABLE 4:** The highest-risk haplotype analysis of *ALDH1A1* in association with PD.

SNP, n	Haplotype^a^	Control, *n* (%)	PD, *n* (%)	*P*	OR (95% CI)
6	GGCTAG	6.4 (0.6)	33.5 (3.3)	8.540 × 10^–6^***	5.619 (2.397–13.172)
5	GG-TAG	6.7 (0.6)	38.1 (3.8)	8.840 × 10^–7^***	6.181 (2.710–14.095)
4	GG-TA-	7.5 (0.7)	44.8 (4.4)	9.610 × 10^–8^***	6.420 (2.944–13.998)
3	GG-T–	17.5 (1.6)	49.2 (4.9)	3.690 × 10^–5^***	3.037 (1.748–5.278)
2	G–T–	320.9 (30.1)	373.6 (36.9)	1.014 × 10^–3^**	1.358 (1.131–1.631)

^*a*^Order of the alleles of the haplotypes: rs4646547, rs1888202, rs348471, rs7043217, rs647880, and rs8187876. A hyphen indicates the eliminated SNP at that position. Haplotypes with frequency < 3% in both PD patients and controls were excluded from the analysis; **P < 0.01; ***P < 0.001. CI, confidence interval; OR, odds ratio; PD, Parkinson’s disease; SNP, single nucleotide polymorphism.

### Interaction Analysis of Variants of *ALDH1A1* and rs4767944 of *ALDH2*

We have previously discovered that the rs4767944 variant of *ALDH2* is associated with PD risk (Zhang et al., 2015), where samples of a total of 766 subjects were also used in the current study, including 402 controls and 364 PD patients. To characterize whether *ALDH1A1* and *ALDH2* were genetically interplayed, this overlapped subgroup was extracted to analyze the interaction between rs7043217 and rs4767944. Results showed that a simultaneous presence of the CC genotype of rs7043217 and the TT genotype of rs4767944 conferred a strong protection against PD (*P* = 4.68 × 10^–4^, OR = 0.378, 95% CI = 0.219–0.652; Table 5).

**TABLE 5:** Interaction analysis between rs7043217 of *ALDH1A1* and rs4767944 of *ALDH2* on risk for PD.

rs7043217	rs4767944	Control, *n* (%)	PD, *n* (%)	*P*^a^	OR (95% CI)
CT + TT	CC + CT	151(37.6)	181(49.7)	Reference	Reference
CT + TT	TT	98(24.4)	86(23.6)	0.177	0.753 (0.499–1.137)
CC	CC + CT	93(23.1)	66(18.1)	0.011*	0.574 (0.374–0.882)
CC	TT	60(14.9)	31(8.5)	4.68 × 10^–4^***	0.378 (0.219–0.652)

^*a*^Adjusted with age and sex; *P < 0.05; ***P < 0.001. CI, confidence interval; OR, odds ratio; PD, Parkinson’s disease.

We also performed the interaction analysis between the other five tag-SNPs of *ALDH1A1* and rs4767944 of *ALDH2*. Since these five tag-SNPs were not significantly associated with PD, both recessive and dominant models were used. Results showed that the higher interaction was present between rs348471 and rs4767944, where the TT genotype of rs348471 and the CC + TT genotypes of rs4767944 led to an aggravated PD risk (*P* = 0.005, OR = 1.930, 95% CI = 1.220–3.055; Supplementary Tables 3, 4).

### *ALDH1A1* mRNA Levels in Peripheral Blood of PD Patients and Controls

It was previously reported that reduced mRNA expression of *ALDH1A1* was a potential blood biomarker of PD (Molochnikov et al., 2012). To replicate this finding, we analyzed *ALDH1A1* mRNA levels in peripheral blood of 19 PD patients and 23 controls with sex and age being matched (*P* > 0.05). Results of real-time PCR showed a trend of decrease in *ALDH1A1* mRNA levels in the PD patients (*P* = 0.084; Supplementary Figure 2).

## Discussion

Animal studies have provided strong evidence linking ALDH1 to PD by dissecting ALDH1-positive neuronal populations in the substantia nigra (Liu et al., 2014). However, it remains elusive whether ALDH1 is genetically associated with PD in humans. By systematically analyzing six tag-SNPs of the ALDH1-encoding gene *ALDH1A1* in a Han Chinese population, we demonstrate that the SNP rs7043217 and the haplotypes of GGCTAG and GGTA are genetically associated with PD susceptibility.

The current study represents the first effort in understanding the genetic association between *ALDH1A1* and PD. Although ALDH1 can catalyze DOPAL oxidation in the brain, the best-known function of this enzyme is to regulate alcohol metabolism. Indeed, genetic variants of *ALDH1A1* have been reported in the context of alcohol drinking. For instance, compared to its T allele, the C allele of rs7043217 is associated with alcohol metabolism capacity in European Americans (Sherva et al., 2009). The G allele of rs610529, which is in linkage disequilibrium with the C allele of rs7043217, is also related to the development of alcohol addiction (Lind et al., 2008). Since a low activity of ALDH protects individuals from alcohol dependence (Luczak et al., 2006), the above results indicate that the C allele of rs7043217 may be associated with high or normal expression or activity of ALDH1. In line with the observation of increased PD risk carrying the rs7043217/T allele, the low ALDH1 activity could lead to DOPAL accumulation in the brain and promote PD pathogenesis (Marchitti et al., 2007).

The association of rs7043217 with overall PD is present in both genotype and allele frequencies. A further PD subtype analysis suggests such an association of rs7043217 is in genotype frequency of the tremor-dominant subtype, and in allele frequency of the postural instability/gait difficulty subtype. Common genetic models include the dominant, recessive, additive, over-dominant, and multiplicative models. All of them are genotype-based, suggesting that genotype frequencies are most commonly used in genetic association studies (Dorak, 2017). However, under multiplicative and additive genetic models, analyzing by allele frequencies provides a more powerful method of testing, suggesting that the allele frequency may be a better indicator in these models (Lewis, 2002). Therefore, both genotype and allele frequencies could serve as an important indicator for certain genetic associations.

It is known that haplotypes are more powerful than individual variants in suggesting genetic association (Gabriel et al., 2002). Indeed, analysis of the six tag-SNPs identifies two haplotypes greatly associated with PD: the GGCTAG serving as a risk factor and the GCCCAG as a protective factor. Further analysis of the effect SNPs suggests that the GGTA of rs4646547, rs1888202, rs7043217, and rs647880 is the highest ranked risk haplotype to discriminate PD, indicating a potential value for genetic diagnosis of PD. Further validation is needed in other populations.

After searching genome-wide association study data, we found a marginal *P* value, 0.058, of rs7043217 in association with PD (Nalls et al., 2019). Given the ALDH is a type of metabolic enzymes, the discrepancy of significance between the studies may result from confounding factors such as differential environmental exposures and interactions with other genetic susceptibilities. For instance, ALDH is susceptible to the inhibition of pesticides. A clade of *ALDH2* haplotypes is shown to exacerbate the risk of PD predisposition by pesticide exposure, but *ALDH2 per se* is not associated with this disorder (Fitzmaurice et al., 2014). Indeed, there are severe abuses of pesticides in China, especially in the East (Jin and Zhou, 2018; Yu et al., 2020), which thus may contribute to the elevated association between rs7043217 of *ALDH1A1* and PD in our cohort. Unfortunately, a lack of pesticide exposure history impedes us from further assessment in this regard. In addition, mice deficient in both *Aldh1a1* and *Aldh2* exhibit age-dependent loss of dopaminergic neurons in the SNpc and motor dysfunctions (Wey et al., 2012). In contrast, genetic deletion of *Aldh1a1* in mice fails to reproduce any overt motor symptoms or loss of dopaminergic neuron in SNpc (Anderson et al., 2011), while no data regarding mice deficient in *Aldh2 per se* are available. These results indicate that ALDH1 and ALDH2 may collectively aggravate PD pathogenesis. Such an interplay is also suggested by our genetic interaction analysis between the tag-SNPs of *ALDH1A1* and rs4767944 of *ALDH2*, with the highest interplay present in the variant rs7043217. The obtained *ALDH1A1* genetic variations thus do not monogenically cause PD.

Besides in the substantia nigra of PD patients (Galter et al., 2003; Grunblatt et al., 2004; Werner et al., 2008), the mRNA expression of *ALDH1A1* is also shown to be reduced in the peripheral blood of sporadic PD patients, and the gene is classified as an optimal predictor for PD risk in a panel with four other genes (Molochnikov et al., 2012). Our results appeared to be in support of such a reduction in PD patients. Nonetheless, a further confirmation in large sample size may be needed. Considering its protective effects on dopaminergic neurons (Liu et al., 2014), the reduced expression of *ALDH1A1* may be a cause rather than a consequence to PD. The associated mechanism likely involves its detoxicating catalyzation against aldehyde risk factors. We herein disclose a genetic association between *ALDH1A1* and PD, represented by the tag-SNP rs7043217. However, all of the 19 SNPs in its linkage disequilibrium block (Supplementary Figure 1) are either located in introns of *ALDH1A1* or a synonymous polymorphism in an exon. It remains to be further determined whether certain SNPs in this block and the associated haplotypes may functionally alter the *ALDH1A1* expression or ALDH1 activity. Indeed, one of the limitations of genetic association studies is that the identified SNPs may not be causative or functional, but they sometimes represent the only practical approach to begin to address a particular biological hypothesis (Hegele, 2002).

In conclusion, the current study demonstrates that PD susceptibility in Han Chinese is associated with *ALDH1A1* genetic variations, notable examples of which include the tag-SNP rs7043217 and the haplotypes GGCTAG and GGTA. These findings provide genetic insights into the role of ALDH1 in PD pathogenesis, and facilitate the development of screening strategies targeting the alleles susceptible to PD for early control of the disease.

## Data Availability Statement

The original contributions presented in the study are publicly available. This data can be found here: https://datadryad.org/stash/share/iPw_jVzOJqOBMAtM_fGeH-TpxEwjB1aV93LU3eBugh8.

## Ethics Statement

The studies involving human participants were reviewed and approved by Ethics Committee of The Second Affiliated Hospital and Yuying Children’s hospital, Wenzhou Medical University. The patients/participants provided their written informed consent to participate in this study.

## Author Contributions

J-HZ and XZ designed the research. H-HF, QG, JZ, and S-SH conducted the experiments. H-HF, Y-ZL, and YS analyzed the data. XZ and MZ contributed to the samples. H-HF and J-HZ wrote the manuscript. All authors have read and approved the final manuscript.

## Conflict of Interest

The authors declare that the research was conducted in the absence of any commercial or financial relationships that could be construed as a potential conflict of interest.

## Footnotes

1 http://analysis.bio-x.cn/SHEsisMain.htm
