# Supplementary material for: ALDH1A1 Genetic Variations May Modulate Risk of Parkinson’s Disease in Han Chinese Population
Source: Front Neurosci. 2021 Mar 19;15:620929. doi: 10.3389/fnins.2021.620929 (PMC8017280; doi:10.3389/fnins.2021.620929)
Supplement: Supplementary file 1 [file Table_1.docx]

**Table S1. Primers and restriction enzymes used in this study**

| **Name** | **Primer** | | **Restriction enzyme** | |
| --- | --- | --- | --- | --- |
| **Genotyping** |  |  |  | |
| rs4646547 | F: | GCAGACCCCAGTGTGTTTGA | MspI | |
|  | R: | GCTCTTGGGCTTTTTGCAGT |  |  |
| rs1888202 | F: | GCCAGAAACGAACCAACTGC | - | |
|  | R: | CATGTGGTATGTTGAGGGGCT |  |  |
| rs348471 | F: | TCCACACTCACAATCCTTGGC | ScaI | |
|  | R: | AAACACAGATCGTAAACTTGCTCA |  |  |
| rs7043217 | F: | GAGTAATAAGCCTTTCCTACTGAC | HinfI | |
|  | R: | GCAGCCTAAGTTAAGGGTTTGT |  |  |
| rs647880 | F: | CCCACTTTAGCCCACAGACT | HinfI | |
|  | R: | CCCACAAACAGAGGCGTTTC |  |  |
| rs8187876 | F: | TGGGGATGATAAAGGCACG | Hpych4IV | |
|  | R: | CAGCATTGGCAGTGTAGCT | |  |
| **Real-time PCR** |  |  | |  |
| *ALDH1A1* | F: | GCACGCCAGACTTACCTGTC | |  |
|  | R: | CCTCCTCAGTTGCAGGATTAAAG | |  |
| *ACTB* | F: | TGGCACCCAGCACAATGAA | |  |
|  | R: | CTAAGTCATAGTCCGCCTAGAAGCA | |  |

F, forward; R, reverse.

**Table S2. Genotype and allele frequencies of the six *ALDH1A1* tag-SNPs in patients of PD subtypes and controls^a^**

| **Tag-SNPs** | **Genotype, n (%)** | | | ***P*^b^** | **Allele, n (%)** | | ***P*^b^** | **OR (95% CI)** |
| --- | --- | --- | --- | --- | --- | --- | --- | --- |
| **rs4646547** | **TT** | **TG** | **GG** |  | **T** | **G** |  |  |
| Control | 103 (19.3) | 276 (51.8) | 154 (28.9) |  | 482 (45.2) | 584 (54.8) |  |  |
| TD | 38 (16.7) | 121 (53.3) | 68 (30.0) | 0.735 | 197 (43.4) | 257 (56.6) | 0.763 | 1.036 (0.822-1.306) |
| PIGD | 25 (16.1) | 81 (52.3) | 49 (31.6) | 0.566 | 131 (42.3) | 179 (57.7) | 0.347 | 1.133 (0.873-1.470) |
| Indeterminate | 4 (12.1) | 20 (60.6) | 9 (27.3) | 0.524 | 28 (42.4) | 38 (57.6) | 0.724 | 1.096 (0.660-1.819) |
| **rs1888202^c^** | **GG** | **GC** | **CC** |  | **G** | **C** |  |  |
| Control | 168 (31.5) | 263 (49.3) | 102 (19.1) |  | 599 (56.2) | 467 (43.8) |  |  |
| TD | 73 (32.2) | 122 (53.7) | 32 (14.1) | 0.251 | 268 (59.0) | 186 (41.0) | 0.483 | 0.920 (0.729-1.161) |
| PIGD | 54 (34.8) | 69 (44.5) | 32 (20.6) | 0.694 | 177 (57.1) | 133 (42.9) | 0.903 | 0.984 (0.758-1.277) |
| Indeterminate | 10 (30.3) | 17 (51.5) | 6 (18.2) | 0.929 | 37 (56.1) | 29 (43.9) | 0.826 | 1.058 (0.638-1.755) |
| **rs348471** | **CC** | **CT** | **TT** |  | **C** | **T** |  |  |
| Control | 143 (26.8) | 288 (54.0) | 102 (19.1) |  | 574 (53.8) | 492 (46.2) |  |  |
| TD | 59 (26.0) | 117 (51.5) | 51 (22.5) | 0.581 | 235 (51.8) | 219 (48.2) | 0.566 | 1.070 (0.850-1.346) |
| PIGD | 48 (31.0) | 67 (43.2) | 40 (25.8) | 0.054 | 163 (52.6) | 147 (47.4) | 0.891 | 1.018 (0.786-1.318) |
| Indeterminate | 11 (33.3) | 17 (51.5) | 5 (15.2) | 0.724 | 39 (59.1) | 27 (40.9) | 0.445 | 0.820 (0.492-1.365) |
| **rs7043217** | **CC** | **CT** | **TT** |  | **C** | **T** |  |  |
| Control | 199 (37.3) | 235 (44.1) | 99 (18.6) |  | 633 (59.4) | 433 (40.6) |  |  |
| TD | 57 (25.1) | 127 (55.9) | 43 (18.9) | 0.007** | 241 (53.1) | 213 (46.9) | 0.061 | 1.247 (0.989-1.572) |
| PIGD | 46 (29.7) | 71 (45.8) | 38 (24.5) | 0.120 | 163 (52.6) | 147 (47.4) | 0.032* | 1.329 (1.025-1.722) |
| Indeterminate | 7 (21.2) | 17 (51.5) | 9 (27.3) | 0.203 | 31 (47.0) | 35 (53.0) | 0.069 | 1.594 (0.964-2.636) |
| **rs647880** | **AA** | **AG** | **GG** |  | **A** | **G** |  |  |
| Control | 135 (25.3) | 261 (49.0) | 137 (25.7) |  | 531 (49.8) | 535 (50.2) |  |  |
| TD | 53 (23.3) | 121 (53.3) | 53 (23.3) | 0.530 | 227 (50.0) | 227 (50.0) | 0.918 | 0.988 (0.785-1.243) |
| PIGD | 46 (29.7) | 74 (47.7) | 35 (22.6) | 0.433 | 166 (53.5) | 144 (46.5) | 0.193 | 0.842 (0.650-1.091) |
| Indeterminate | 11 (33.3) | 14 (42.4) | 8 (24.2) | 0.616 | 36 (54.5) | 30 (45.5) | 0.446 | 0.822 (0.497-1.360) |
| **rs8187876** | **GG** | **GA** | **AA** |  | **G** | **A** |  |  |
| Control | 149 (28.0) | 269 (50.5) | 115 (21.6) |  | 567 (53.2) | 499 (46.8) |  |  |
| TD | 48 (21.1) | 126 (55.5) | 53 (23.3) | 0.144 | 222 (48.9) | 232 (51.1) | 0.133 | 1.193 (0.948-1.502) |
| PIGD | 48 (31.0) | 79 (51.0) | 28 (18.1) | 0.461 | 175 (56.5) | 135 (43.5) | 0.229 | 0.853 (0.658-1.105) |
| Indeterminate | 8 (24.2) | 17 (51.5) | 8 (24.2) | 0.890 | 33 (50.0) | 33 (50.0) | 0.638 | 1.128 (0.683-1.862) |

^a^ PD subtypes were respectively compared to the controls; ^b^ Adjusted with age and sex; ^c^ rs1888202 genotyped by Sanger sequencing, and the other 5 SNPs genotyped by polymerase chain reaction-restriction fragment length polymorphism; *, *P* < 0.05; **, *P* < 0.01.

CI, confidence interval; OR, odds ratio; PD, Parkinson’s disease; PIGD, postural instability/gait difficulty; SNP, single nucleotide polymorphism; TD, tremor dominant

**Table S3.** **Interaction analysis between the other 5 tag-SNPs of *ALDH1A1* (using a recessive model) and rs4767944 of *ALDH2* on risk for PD**

| **SNP1** | **SNP2** | **Control, n (%)** | **PD, n (%)** | ***P*^a^** | **OR (95% CI)** |
| --- | --- | --- | --- | --- | --- |
| **rs4646547** | **rs4767944** |  |  |  |  |
| TG + TT | CC + CT | 171 (42.5) | 167 (45.9) | reference | reference |
| TG + TT | TT | 107 (26.6) | 78 (21.4) | 0.133 | 0.734 (0.490-1.099) |
| GG | CC + CT | 73 (18.2) | 80 (22.0) | 0.740 | 1.077 (0.697-1.664) |
| GG | TT | 51 (12.7) | 39 (10.7) | 0.268 | 0.738 (0.432-1.262) |
| **rs1888202** | **rs4767944** |  |  |  |  |
| GC + GG | CC + CT | 199 (49.5) | 207 (56.9) | reference | reference |
| GC + GG | TT | 123 (30.6) | 94 (25.8) | 0.090 | 0.724 (0.499-1.052) |
| CC | CC + CT | 45 (11.2) | 40 (11.0) | 0.974 | 1.009 (0.593-1.717) |
| CC | TT | 35 (8.7) | 23 (6.3) | 0.288 | 0.704 (0.369-1.344) |
| **rs348471** | **rs4767944** |  |  |  |  |
| CT + CC | CC + CT | 187 (46.5) | 166 (45.6) | reference | reference |
| CT + CC | TT | 115 (28.6) | 84 (23.1) | 0.194 | 0.770 (0.519-1.142) |
| TT | CC + CT | 57 (14.2) | 81 (22.3) | 0.005** | 1.930 (1.220-3.055) |
| TT | TT | 43 (10.7) | 33 (9.1) | 0.542 | 1.199 (0.669-2.147) |
| **rs647880** | **rs4767944** |  |  |  |  |
| AG + AA | CC + CT | 183 (45.5) | 199 (54.7) | reference | reference |
| AG + AA | TT | 117 (29.1) | 86 (23.6) | 0.098 | 0.718 (0.485-1.064) |
| GG | CC + CT | 61 (15.2) | 48 (13.2) | 0.237 | 0.745 (0.458-1.213) |
| GG | TT | 41 (10.2) | 31 (8.5) | 0.046* | 0.563 (0.321-0.990) |
| **rs8187876** | **rs4767944** |  |  |  |  |
| GA + GG | CC + CT | 187 (46.5) | 193 (53.0) | reference | reference |
| GA + GG | TT | 126 (31.3) | 89 (24.5) | 0.061 | 0.694 (0.474-1.017) |
| AA | CC + CT | 57 (14.2) | 54 (14.8) | 0.975 | 0.992 (0.614-1.603) |
| AA | TT | 32 (8.0) | 28 (7.7) | 0.496 | 0.809 (0.439-1.489) |

^a^ Adjusted with age and sex; *, *P* < 0.05; **, *P* < 0.01.

CI, confidence interval; OR, odds ratio; PD, Parkinson’s disease

**Table S4.** **Interaction analysis between the other 5 tag-SNPs of *ALDH1A1* (using a dominant model) and rs4767944 of *ALDH2* on risk for PD**

| **SNP1** | **SNP2** | **Control, n (%)** | **PD, n (%)** | ***P*^a^** | **OR (95% CI)** |
| --- | --- | --- | --- | --- | --- |
| **rs4646547** | **rs4767944** |  |  |  |  |
| TG + GG | CC + CT | 197 (49.0) | 212 (58.2) | reference | reference |
| TG + GG | TT | 133 (33.1) | 97 (26.6) | 0.043* | 0.684 (0.474-0.988) |
| TT | CC + CT | 47 (11.7) | 35 (9.6) | 0.171 | 0.679 (0.390-1.182) |
| TT | TT | 25 (6.2) | 20 (5.5) | 0.191 | 0.626 (0.310-1.264) |
| **rs1888202** | **rs4767944** |  |  |  |  |
| GC + CC | CC + CT | 166 (41.3) | 173 (47.5) | reference | reference |
| GC + CC | TT | 112 (27.9) | 86 (23.6) | 0.097 | 0.714 (0.479-1.063) |
| GG | CC + CT | 78 (19.4) | 74 (20.3) | 0.166 | 0.733 (0.473-1.137) |
| GG | TT | 46 (11.4) | 31 (8.5) | 0.024* | 0.518 (0.293-0.916) |
| **rs348471** | **rs4767944** |  |  |  |  |
| CT + TT | CC + CT | 195 (48.5) | 200 (54.9) | reference | reference |
| CT + TT | TT | 128 (31.8) | 93 (25.5) | 0.080 | 0.715 (0.491-1.041) |
| CC | CC + CT | 49 (12.2) | 47 (12.9) | 0.589 | 0.870 (0.524-1.443) |
| CC | TT | 30 (7.5) | 24 (6.6) | 0.177 | 0.641 (0.336-1.223) |
| **rs647880** | **rs4767944** |  |  |  |  |
| AG + GG | CC + CT | 179 (44.5) | 184 (50.5) | reference | reference |
| AG + GG | TT | 121 (30.1) | 85 (23.4) | 0.045* | 0.671 (0.455-0.990) |
| AA | CC + CT | 65 (16.2) | 63 (17.3) | 0.830 | 1.051 (0.665-1.663) |
| AA | TT | 37 (9.2) | 32 (8.8) | 0.845 | 0.942 (0.520-1.708) |
| **rs8187876** | **rs4767944** |  |  |  |  |
| GA + AA | CC + CT | 166 (41.3) | 183 (50.3) | reference | reference |
| GA + AA | TT | 124 (30.8) | 92 (25.3) | 0.072 | 0.702 (0.478-1.032) |
| GG | CC + CT | 78 (19.4) | 64 (17.6) | 0.917 | 0.625 (0.625-1.525) |
| GG | TT | 34 (8.5) | 25 (6.9) | 0.407 | 0.402 (0.402-1.446) |

^a^ Adjusted with age and sex; *, *P* < 0.05.

CI, confidence interval; OR, odds ratio; PD, Parkinson’s disease


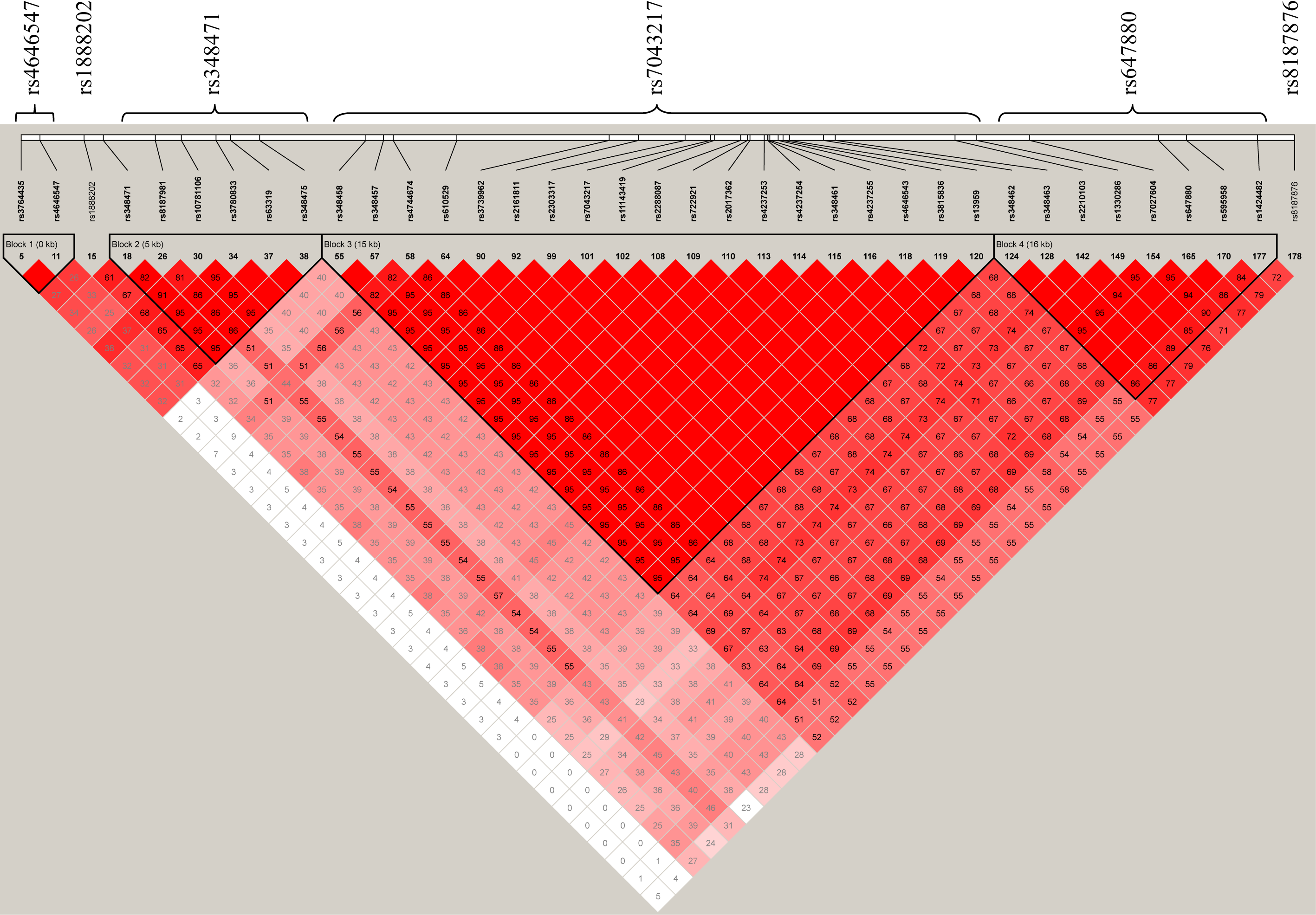


**Fig. S1. View of SNPs and the six tag-SNPs in *ALDH1A1* gene.** Rs4646547 represents the linkage disequilibrium block 1, rs348471 represents block 2, rs7043217 represents block 3 and rs647880 represents the block 4. SNP, single nucleotide polymorphism.


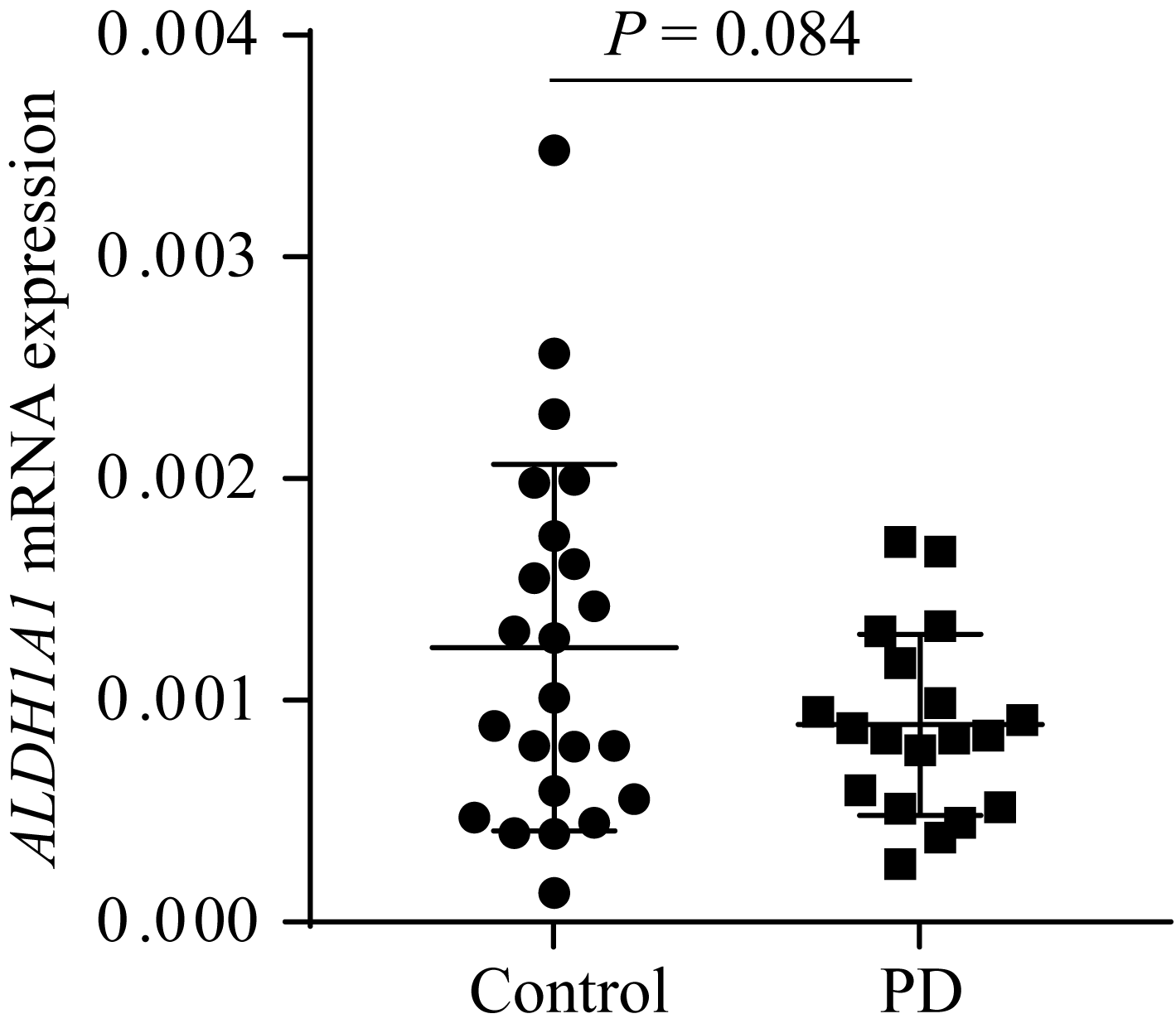


**Fig. S2. The mRNA levels of *ALDH1A1* in peripheral blood.** n **=** 19 and 23, respectively for PD patients and controls. PD, Parkinson’s disease.
